# Psychological Hardiness and Burnout in the Context of Post-Traumatic Stress Disorder Among South African First Responders

**DOI:** 10.1177/24705470251373021

**Published:** 2025-08-25

**Authors:** Anita Padmanabhanunni, Tyrone B Pretorius

**Affiliations:** 1 Department of Psychology, 56390 University of the Western Cape, Cape Town, South Africa

**Keywords:** PTSD, hardiness, commitment, challenge, control, emotional exhaustion, depersonalization

## Abstract

Abstract

**Background:**

Exposure to traumatic events is an inherent aspect of first responder work, placing individuals at heightened risk for post-traumatic stress disorder (PTSD) and burnout. This study examined the relationship between PTSD symptoms and two key dimensions of burnout—emotional exhaustion and depersonalization—among South African first responders, with a particular focus on the mediating role of psychological hardiness.

**Methods:**

A total of 429 participants (police officers and paramedics) completed the PTSD Checklist for DSM-5 (PCL-5), the Short Hardiness Scale, and the Emotional Exhaustion and Depersonalization subscales of the Maslach Burnout Inventory.

**Results:**

Path analysis revealed that the control and challenge dimensions of hardiness partially mediated the relationship between PTSD and burnout. While higher control was associated with lower burnout, higher challenge was unexpectedly associated with greater burnout. This suggests that different hardiness dimensions play distinct roles in the PTSD–burnout relationship. In contrast, the commitment dimension did not mediate this relationship.

**Conclusion:**

These findings highlight the nuanced and multidimensional role of hardiness in trauma-exposed populations and underscore the importance of resilience-focused interventions that enhance perceived control and constructive engagement with challenge to mitigate burnout in high-risk occupational groups.

## Introduction

Post-traumatic stress disorder (PTSD) and burnout are significant public health concerns among first responders—such as police officers, paramedics, firefighters, and other emergency service personnel.^1^ They are routinely exposed to potentially traumatic events in the course of their work, including violent incidents, fatal accidents, natural disasters, and human suffering.^1,2^ Unlike the general population, where exposure to trauma may be infrequent, first responders face repeated and cumulative exposure to such events as a normative aspect of their professional roles. Over time, this high level of occupational stress can lead to profound psychological consequences and place them at elevated risk of experiencing mental health disorders.^2,3^

PTSD in first responders is characterized by intrusive memories, avoidance behaviors, negative alterations in cognition and mood, and hyperarousal, all of which can impair occupational functioning and quality of life.^1^ Recent reviews have attested to the prevalence of PTSD and other mental health disorders among first responders. For example, a scoping review reported a PTSD prevalence rate of 57% among firefighters.^4^ Similarly, Wagner and colleagues^5^ found prevalence rates for PTSD among firefighters ranging from 6.4% to 57%, while Syed, Ashwick^6^ reported rates between 5.8% and 19.6% among police officers. In a meta-analysis focused on ambulance personnel, Petrie and colleagues^7^ estimated prevalence rates of 11% for PTSD, 15% for depression, 15% for anxiety, and 27% for general psychological distress.

In contrast to PTSD, burnout manifests as emotional exhaustion, depersonalization, and a reduced sense of personal accomplishment.^8^ Emotional exhaustion reflects the core component of burnout and is characterized by a feeling of being overextended and emotionally drained. Individuals experiencing emotional exhaustion often feel they have nothing left to give emotionally and may struggle to cope with daily demands. Depersonalization, also referred to as cynicism or detachment, involves developing a distant, indifferent, or negative attitude toward one's work or the people one serves.^9^ It involves a state of apathy in which first responders adopt an uncaring attitude so as to protect themselves from occupational stress. Burnout has substantive consequences in terms of job performance and can result in high turnover rates, absenteeism, impaired decision-making and decreased productivity and efficiency.^10^

While distinct, PTSD and burnout can co-occur, with shared risk factors including chronic stress, inadequate organizational support, long working hours, and stigma around help-seeking. Despite the well-documented risk, mental health issues among first responders are frequently under-recognized and under-treated. Structural and cultural barriers within emergency services, such as stoicism, stigma, and prioritization of operational readiness, can hinder timely identification and intervention.^10,11^

Despite repeated occupational exposure to potentially traumatic events, many first responders do not experience mental health problems.^12^ This highlights the need to better understand the factors that contribute to differential vulnerability. This study contributes to this goal by investigating the role of hardiness in the relationship between PTSD and two components of burnout, namely emotional exhaustion and depersonalization. The construct of hardiness represents a set of protective personality traits that enhance an individual's resilience in the face of stress and adversity.^13^

Hardiness, first conceptualized by Kobasa.^14^ is composed of three interrelated components namely commitment, control and challenge. While commitment refers to the tendency to remain actively engaged in life's activities and to see challenges as meaningful rather than burdensome, control entails the belief that one can influence events and outcomes in their life through effort and action. Challenge involves appraising change and difficulty not as threats, but as opportunities for growth and learning.^15^ Together, these traits form a psychological buffer that promotes adaptive coping and reduces vulnerability to stress-related outcomes such as burnout and mental health disorders. Hardiness has been shown to moderate the effects of stress by fostering persistence, optimism, and problem-solving under pressure. For instance, Bartone and colleagues^13^ found that while COVID-19 stress was associated with elevated symptoms of anxiety and depression, individuals with high levels of hardiness reported significantly lower levels of these symptoms. Similarly, a study of severely wounded military personnel and their spouses found that those high in hardiness were more likely to experience psychological wellbeing following extremely stressful experiences.^16^

Hardiness has been shown to function as an important psychological mechanism in mitigating the adverse impact of stress and trauma on mental health. For instance, it has been identified as a significant moderator in the relationship between workaholism and occupational burnout among nurses.^17^ In military populations, hardiness has been linked to improved outcomes under stress.^18^ A study of army reservists found that psychological hardiness positively influenced performance under high stress, with its effects moderated by stress levels and personal resilience.^18^ Empirical evidence has also supported its mediating role across diverse contexts. For example, it partially mediated the association between perceived stress and happiness among nurses,^19^ the relationship between perceived loneliness and depressive symptoms among older people^20^ as well as between social support and optimal outcomes such as lower depression, better self-reported health, and higher life satisfaction in the elderly.^21^ This body of work suggests that hardiness not only confers resilience in the face of adverse experiences but also operates as a psychological pathway through which stress-related exposures influence mental health outcomes.

In the current study, we extended this line of inquiry to the context of first responders, a group frequently exposed to potentially traumatic events and at elevated risk for both PTSD and burnout. Specifically, our model posits that PTSD symptoms, reflecting both trauma exposure and associated psychological distress, affect burnout outcomes (emotional exhaustion and depersonalization) both directly and indirectly through the dimensions of hardiness. By testing this mediation pathway, we aimed to clarify the mechanisms through which posttraumatic symptomatology contributes to burnout, and to identify whether certain dimensions of hardiness are more influential than others in this relationship. This model structure was chosen to align with prior evidence of hardiness as a mediator and to provide an understanding of how individual resilience traits may attenuate the negative occupational consequences of PTSD in high-risk professions.

This suggests that hardiness can buffer the negative psychological effects of stressful events. For the current study, we hypothesized that:

- Higher levels of PTSD symptoms will be positively associated with greater emotional exhaustion and depersonalization among first responders.
- The hardiness dimensions of challenge, control, and commitment will be negatively associated with emotional exhaustion and depersonalization.
- The hardiness dimensions of challenge, control, and commitment will mediate the relationship between PTSD symptoms and both emotional exhaustion and depersonalization.

## Materials and Method

### Participants and Procedure

The current study was cross-sectional in nature and adhered to the STROBE reporting guidelines for such studies. Participants were first responders (*n* = 429) from the Western Cape province of South Africa, comprising police officers (*n* = 309) and paramedics (*n* = 120). Participants were eligible for inclusion if they were currently employed as first responders in the Western Cape province of South Africa, specifically in the roles of police officer or paramedic, were aged 18 years or older, and provided informed consent to participate. Individuals were excluded if they were not actively employed as first responders in the province at the time of data collection (eg, retired, unemployed, volunteers, or employed in other roles such as firefighters or emergency medical technicians), if they declined or withdrew consent to participate.

An electronic version of the questionnaire, developed using Google Forms, was distributed online via Facebook groups dedicated to first responders—permission to post the survey link and participation invitation was obtained from group administrators. In addition, formal approval was secured from the South African Police Services (Reference: 3/34/2, 27 June 2023) and the Western Cape Department of Health (Reference: WC_202307_041, 15 September 2023). Research assistants also visited police stations and hospitals to recruit participants in person.

The socio-demographic characteristics of the sample are summarized in Table 1. The majority of participants were male (55%) and worked in urban settings (92.3%). Educational attainment was nearly evenly split between those holding a National Senior Certificate (49.2%) and those with post-secondary qualifications (49.65%). Slightly more than half of the participants were married (51.5%). The mean age was 39.0 years (*SD* = 9.9), and the mean duration of service as a first responder was 13.2 years (*SD* = 9.7).

**Table 1.** Description of the Sample.

Variable	Groups	*n/X̄*	%/*SD*
Gender	Female	193	45%
	Male	236	55%
First responder category	Police officer	309	72%
	Paramedic	120	28%
Highest Qualification	< Grade 12	5	1.2%
	National Senior Certificatea	211	49.2%
	Post Grade 12 Diploma/certificate	145	33.8%
	Undergraduate degree	41	9.6%
	Postgraduate degree	27	6.3%
Work setting	Urban	396	92.3%
	Peri-Urban	11	2.6%
	Rural	22	5.1%
Relationship status	Single	151	35.2%
	Married	221	51.5%
	Divorced/separated	57	13.3
Age	N/A	39.0 years	9.9
Length of service	N/A	13.2 years	9.7

*Note:* ^a^Grade 12

### Measures

As part of a broader study on the mental health of first responders, participants completed the standardized instruments detailed below. The internal consistency reliability of the instruments as used in the current study can be found in Table 2.

- Post-Traumatic Stress Disorder Checklist for DSM-5 (PCL-5; Blevins et al, 2015)
  The PCL-5 is a 20-item scale that assesses the presence and severity of PTSD symptoms using a 5-point Likert scale ranging from 0 (*not at all*) to 4 (*extremely*). An example item is: “How much were you bothered by feeling very upset when someone reminded you of the stressful experience?” The scale has demonstrated excellent internal consistency (α = .95) and sound convergent and discriminant validity.^22^ South African studies have reported similar reliability estimates (eg,^23^: α and ω = .94).
- Short Hardiness Scale (SHS: 15)
  The SHS consists of 15 items measuring three dimensions of psychological hardiness: commitment, challenge, and control (5 items each). Example items include: “Most of my life is spent doing things that are worthwhile” (commitment), “Changes in routine are interesting to me” (challenge), and “By working hard you can always achieve your goals” (control). Responses are recorded on a 4-point scale from 0 (*not at all true*) to 3 (*completely true*). Reported reliability coefficients range from .70 to .77, with evidence for criterion and predictive validity.^15^
- Maslach Burnout Inventory^9^

**Table 2.** Intercorrelations, Descriptive Statistics, Distribution Indices, and Reliability Estimates.

Variable/Scale	1	2	3	4	5	6
1. PTSD	—					
2. Commitment	−.05	—				
3. Control	−.15*	.73**	—			
4. Challenge	.15*	.70**	.63**	—		
5. Emotional exhaustion	.63**	.07	−.07	.28**	—	
6. Depersonalization	.56**	.13*	−.03	.30**	.80**	—
Mean	31.2	8.4	9.7	8.3	24.9	12.5
SD	17.0	3.1	3.1	3.1	12.6	7.9
Skewness	0.10	−0.04	−0.33	0.24	−0.02	0.10
Kurtosis	−0.49	−0.29	−0.45	−0.32	−0.79	−1.06
Alpha	.95	.68	.76	.73	.90	.84
Omega	.94	.69	.76	.73	.90	.84

*P < .01, **P < .001

This study used the Emotional Exhaustion (EE; 9 items) and Depersonalization (DP; 5 items) subscales, which represent negative indicators of burnout. Example items include: “I feel used up at the end of the workday” (EE) and “I feel I treat some of the people I am meant to serve as if they were impersonal objects” (DP). Responses to the items of the two subscales are made on a seven-point scale ranging from 0 (*never*) to 6 (*every day*). Reported reliability for the EE and DP subscales is .83 and .77, respectively.^9^ In South Africa, acceptable estimates of internal consistency were reported in a study with school teachers (ref.^24^: EE = .94, DP = .83) and with university educators (ref.^25^: EE = .89, DP = .71).

### Ethics

Ethical approval for the study was granted by the Humanities and Social Sciences Research Ethics Committee of the University of the Western Cape (Ethics Reference: HS23/2/4, 23 May 2023) and was conducted in line with the Declaration of Helsinki. Informed electronic consent was obtained on the landing page of the survey. No identifying information was collected from participants.

### Data Analysis

Descriptive statistics (means and standard deviations), distribution indices (skewness and kurtosis), intercorrelations (Pearson's r), and internal consistency estimates (Cronbach's alpha and McDonald's omega) were computed using IBM SPSS for Windows, version 30. Normality was inferred when skewness and kurtosis values fell between −2 and +2.^26^ Internal consistency values ≥ .70 were considered acceptable for research purposes,^27^ while values between .60 and .69 were considered marginally reliable.^28^

Path analysis was conducted in IBM AMOS for Windows, version 28, to test the proposed mediation role of hardiness. In this analysis, PTSD symptoms were specified as the predictor variable, the three hardiness dimensions (commitment, control, and challenge) as parallel mediators, and the two burnout components (emotional exhaustion and depersonalization) as outcome variables. Direct effects represented the associations between PTSD and each burnout component in the presence of the mediators, while indirect effects captured the pathways through each hardiness dimension. The significance of both direct and indirect effects was evaluated using bootstrapped 95% confidence intervals and corresponding *P*-values. To examine the change in the relationship between PTSD symptoms and burnout in the absence and presence of the mediators, separate regression analyses were conducted without the mediators. If the relationship between PTSD and burnout was significant in this separate analysis but non-significant in the mediation analysis it would be indicative of full mediation. On the other hand, if the relationship between PTSD and burnout is reduced but still significant it would indicate partial mediation.

## Results

Descriptive statistics (means and standard deviations), intercorrelations (Pearson's r), distribution indices (skewness and kurtosis), and internal consistency estimates (Cronbach's alpha and McDonald's omega) are reported in Table 2.

Skewness and kurtosis values ranged from −1.06 to 0.24, indicating that all variables were approximately normally distributed. Internal consistency coefficients were above the acceptable threshold of .70 for all scales, except for the commitment dimension, which demonstrated marginal reliability.

Regarding intercorrelations, PTSD was significantly positively associated with emotional exhaustion (*r* = .63, *P* < .001, large effect), depersonalization (*r* = .56, *P* < .001, large effect), and challenge (*r* = .15, *P* = .002, small effect), and negatively associated with control (*r* = −.15, *P* = .002, small effect). These results suggest that higher PTSD scores were linked to higher emotional exhaustion, depersonalization, and challenge, and lower levels of control.

Commitment was positively correlated with depersonalization (*r* = .13, *P* = .007, small effect). The challenge dimension was positively associated with emotional exhaustion (*r* = .28, *P* < .001, small effect) and depersonalization (*r* = .30, *P* < .001, medium effect), indicating that greater perceptions of challenge were related to greater burnout symptoms.

Direct and indirect effects of PTSD on the burnout dimensions are presented in Table 3.

**Table 3.** Direct and Indirect Effects of PTSD on Burnout Components.

Effect	B	SE	95% CI		β	*P*-value
			LL	UL		
Direct effects						
PTSD → Emotional exhaustion	.41	.03	.36	.46	.53	<.001
PTSD → Depersonalization	.23	.02	.20	.26	.48	<.001
Indirect effects						
PTSD → Challenge → Emotional exhaustion	.03	.01	.02	.06	.04	.002
PTSD → Challenge → Depersonalization	.02	.01	.01	.03	.04	.002
PTSD → Commitment → Emotional exhaustion	−.00	.00	−.01	.00	−.00	.326
PTSD → Commitment → Depersonalization	−.00	.00	−.01	.00	−.01	.219
PTSD → Control → Emotional exhaustion	.02	.01	.01	.04	.03	.002
PTSD → Control → Depersonalization	.02	.01	.01	.03	.03	.001

From Table 3 it is evident that:

- The direct effects indicate that PTSD significantly predicted emotional exhaustion (β = .53, *P* < .001) and depersonalization (β = .48, *P* < .001).
- Challenge partially mediated the relationship between PTSD and both emotional exhaustion (β = .04, *P* = .002) and depersonalization (β = .04, *P* = .002), as the direct effects remained significant in the presence of the mediator.
- Control also emerged as a partial mediator in the relationships between PTSD and emotional exhaustion (β = .03, *P* = .002), and between PTSD and depersonalization (β = .03, *P* = .001).
- In contrast, commitment did not significantly mediate the relationship between PTSD and either emotional exhaustion or depersonalization.

A separate regression analysis was conducted without the mediators and it demonstrated that in the absence of the mediator the association between PTSD and emotional exhaustion was β = .63 (*P* < .001) and as can be seen from Table 2, this was reduced to β = .53 (*P* < .001) in the presence of the mediators. Similarly, the association between PTSD and depersonalization was β = .56 (*P* < .001) in the absence of the mediator, and the reduced to β = .48 (*P* < .001) in the presence of the mediators.

**Figure 1.**
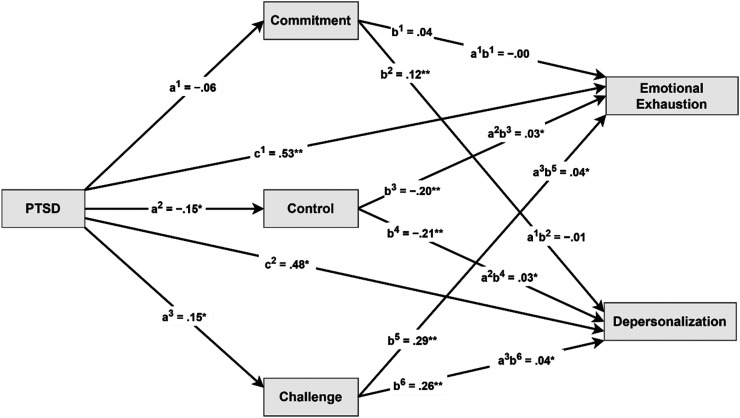
Mediation Model: The Role of Hardiness Dimensions in the Relationship Between PTSD and Burnout. *Note*: c^1^, c2 = direct effects of PTSD on emotional exhaustion and depersonalization; a^1^–a^3^ = direct effects of PTSD on mediators; b^1^–b^6^ = direct effects of mediators on dependent variables; a^1^b^1^, a^1^b^2^ indirect effects of predictor on dependent variables through commitment; a^2^b^3^, a^2^b^4^ = indirect effects of predictor on dependent variable through control; a^3^b^5^, a^3^b^6^ = indirect effects of predictor on dependent variable through challenge; All regression coefficients are standardized. *P < .01, **P < .001.

These mediational pathways are visually represented in Figure 1.

In summary, the findings indicate that challenge and control significantly mediated the impact of PTSD on emotional exhaustion and depersonalization, while commitment did not play a significant mediating role.

## Discussion

This study aimed to investigate the protective role of hardiness in the relationship between PTSD and two dimensions of burnout specifically, emotional exhaustion and depersonalization, among first responders. Several important findings emerged from the analysis. First, higher levels of PTSD were strongly associated with greater emotional exhaustion and depersonalization. This reinforces the well-established link between trauma exposure and the erosion of emotional and psychological resources, particularly in high-stress occupational contexts.^29,30^ Interestingly, PTSD symptoms were also associated with lower levels of the control dimension of hardiness, suggesting that trauma may undermine individuals’ sense of agency and perceived ability to influence outcomes. This perceived lack of control may, in turn, intensify feelings of helplessness and emotional fatigue.

Second, a more complex pattern emerged in relation to the challenge dimension. Although challenge is typically viewed as a protective trait, higher PTSD symptoms were unexpectedly associated with greater perceptions of challenge, which in turn were linked to increased emotional exhaustion and depersonalization. This pattern suggests that in the context of chronic trauma exposure, perceiving challenges as opportunities for growth may no longer serve an adaptive function. Rather than motivating engagement and growth, it may reflect a heightened awareness of ongoing demands that exceed one's coping capacity, thus contributing to burnout. This observation is consistent with prior work on the challenge facet of hardiness. For example, Potard and colleagues^31^ found that, among police officers, a greater tendency to view stressors as challenges did not translate into lower PTSD symptoms. They argued that framing adverse situations in terms of opportunity and mastery may bolster task-related performance without necessarily conferring mental health benefits.

Third, the commitment dimension of hardiness was positively correlated with depersonalization. This counterintuitive finding may reflect a form of overidentification with one's professional role, which, when paired with chronic trauma exposure, could lead to emotional distancing as a self-protective mechanism. It is possible that individuals who remain highly committed to their roles despite experiencing distress may be more vulnerable to emotional withdrawal and detachment from others, particularly when their efforts feel unrecognized or ineffective. This interpretation is supported by previous research examining the role of occupational calling. For instance, a study investigating the relationship between a sense of calling, PTSD, and burnout among firefighters found that those who strongly viewed their work as a calling reported higher levels of both PTSD symptoms and burnout.^32^ The authors suggested that while a sense of calling may initially motivate persistence and dedication, it may also heighten vulnerability to psychological distress when individuals feel unable to live up to internalized ideals or when the work environment fails to support their efforts. This highlights the potential paradoxical role of commitment in high-risk, trauma-exposed professions.

Fourth, mediation analyses revealed that both challenge and control partially mediated the relationships between PTSD symptoms and the two dimensions of burnout, namely emotional exhaustion and depersonalization. These findings underscore the central role that perceptions of control and challenge play in shaping how trauma-related distress translates into burnout. While high challenge and low control may both amplify the risk of burnout in the presence of PTSD symptoms, they also point to potential intervention targets. In contrast, commitment did not significantly mediate the relationship between PTSD symptoms and burnout. This suggests that while commitment may be a valued trait in the workplace, it may not exert a protective effect in trauma-laden contexts, particularly if it is not accompanied by adequate support and psychological resources.

Taken together, these findings suggest that psychological hardiness is a multi-faceted construct whose components function differently under conditions of chronic stress. Interventions aimed at reducing burnout among trauma-exposed populations may benefit from focusing specifically on enhancing control and adaptive engagement with challenge, rather than assuming that all aspects of hardiness are uniformly protective.

The findings of this study highlight several important implications for the development of targeted mental health interventions for first responders. The significant associations between PTSD symptoms and burnout underscore the need for integrated trauma-informed care that addresses both posttraumatic stress and occupational exhaustion.^33^ The mediating roles of control and challenge suggest that bolstering specific dimensions of psychological hardiness may be an effective strategy for reducing burnout. Programmes that enhance perceived control, through training in adaptive coping, problem-solving, and decision-making autonomy, may help first responders feel more capable in managing the demands of their roles.^34^ Similarly, interventions that support constructive engagement with challenging situations (eg, cognitive reframing, goal-setting aligned with capacity) may buffer the effects of trauma exposure on burnout, provided these approaches are balanced with realistic appraisals of workload.

Finally, the complex relationship between commitment and burnout highlights the importance of addressing cultural narratives of overidentification with professional roles. Psychoeducational and reflective practices that promote healthy boundaries, self-compassion, and recognition of emotional limits can help prevent the internalization of unrealistic expectations and reduce the risk of detachment and exhaustion, particularly among highly dedicated personnel.^35^

Several limitations should be considered when interpreting the findings of this study. The use of a cross-sectional design precludes any inference of causality between PTSD symptoms, hardiness dimensions, and burnout outcomes. Longitudinal studies are needed to establish temporal relationships and to better understand how these constructs interact over time. The sample was limited to two categories of first responders, which may restrict the generalizability of the findings to other high-risk professions. All data were obtained through self-report measures, which are subject to potential response biases such as social desirability or underreporting of symptoms. Incorporating objective measures or clinician-administered assessments in future studies may provide a more comprehensive picture of psychological functioning. Finally, the study was conducted within a single geographical region, limiting the applicability of the findings to other contexts. Replication in other regions or countries is essential to confirm the robustness of these associations across diverse settings.

Future research should explore moderating factors that may help explain variability in burnout among trauma-exposed professionals. In particular, organizational support (eg, perceived supervisor support, availability of psychological resources, and supportive workplace policies) may play a critical role in buffering the negative impact of PTSD symptoms on burnout. Similarly, individual coping strategies, including problem-focused and emotion-focused approaches, may influence how first responders manage ongoing occupational stressors and, in turn, their vulnerability to burnout. Examining these factors in conjunction with resilience traits such as hardiness could provide a more comprehensive understanding of the protective and risk mechanisms operating in high-stress occupational contexts.

## Conclusion

This study contributes to a growing body of literature on the psychological challenges faced by first responders by examining the associations between PTSD symptoms, burnout, and psychological hardiness. The findings suggest that while PTSD symptoms significantly predict burnout, the protective effects of hardiness are nuanced. Specifically, higher control was associated with reduced burnout and acted as a partial mediator, whereas higher challenge was unexpectedly linked to greater burnout, indicating that this dimension may not operate adaptively under conditions of chronic trauma exposure. These insights underscore the importance of tailored, evidence-based interventions that strengthen protective traits such as control, while addressing potentially maladaptive patterns in other traits like challenge, within the demanding realities of first responder work.
